# National burden and risk factors of diabetes mellitus in China from 1990 to 2021: Results from the Global Burden of Disease study 2021

**DOI:** 10.1111/1753-0407.70012

**Published:** 2024-10-07

**Authors:** Wenzhen Deng, Li Zhao, Cheng Chen, Ziyu Ren, Yuanyuan Jing, Jingwen Qiu, Dongfang Liu

**Affiliations:** ^1^ Department of Endocrinology The Second Affiliated Hospital of Chongqing Medical University Chongqing China; ^2^ Department of Endocrinology Qianjiang Central Hospital of Chongqing Qianjiang China

**Keywords:** diabetes mellitus, disability‐adjusted life years, global disease burden, incidence, prevalence

## Abstract

**Background:**

In recent years, the prevalence and mortality rates of diabetes have been rising continuously, posing a significant threat to public health and placing a heavy burden on the population. This study was conducted to describe and analyze the burden of diabetes in China from 1990 to 2021 and its attributable risk factors.

**Methods:**

Utilizing data from the Global Burden of Disease Study 2021, we analyzed the incidence, prevalence, and disability‐adjusted life years (DALYs) of type 1 diabetes (T1DM) and type 2 diabetes (T2DM) in China from 1990 to 2021. We extracted sex‐ and age‐specific data on diabetes, focusing on DALYs, years lived with disability, and years of life lost. Bayesian meta‐regression and spatiotemporal Gaussian process regression were used to estimate disease parameters. Age‐standardized rates (ASRs) and estimated annual percentage changes (EAPC) were calculated using direct standardization and log‐linear regression. The population‐attributable fractions were also determined for each risk factor.

**Results:**

In 2021, the absolute number of incident diabetes mellitus (DM) cases was estimated at 4003543.82, including 32 000 T1DM and 3971486.24 T2DM cases. The ASRs were 244.57 for DM, 2.67 for T1DM, and 241.9 for T2DM (per 100 000 population). The absolute number of prevalent DM cases was 117288553.93, including 1442775.09 T1DM and 115845778.84 T2DM cases. The ASRs were 6142.29 for DM, 86.78 for T1DM, and 6055.51 for T2DM (per 100 000 population). In 2021, there were 178475.73 deaths caused by DM, with an ASR of mortality of 8.98 per 100 000 population. The DALYs due to DM in 2021 were 11713613.86, with an ASR of 585.43 per 100 000 population and an EAPC of 0.57. This increase can be attributed to several factors, including high body mass index, air pollution, and dietary habits.

**Conclusions:**

The burden of diabetes is considerable, with high prevalence and incidence rates, highlighting the urgent need for public health interventions. Addressing factors like high fasting plasma glucose, body mass index, air pollution, and dietary risks through effective interventions is critical.

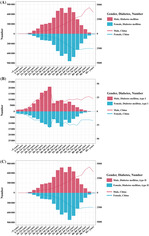

## Graphical abstract

Highlights

- The incidence, prevalence, and DALYs rate of diabetes in China are at relatively high levels, with a severe and rapidly growing burden of disability, particularly prominent among males.
- Interventions should be strengthened for major factors contributing to the diabetes burden, including high fasting plasma glucose, high body mass index, air pollution, and dietary risks.

## 1 BACKGROUND

Diabetes mellitus (DM) is one of the leading chronic noncommunicable diseases, with its prevalence and mortality rates steadily increasing in recent years.^1^ This rise poses a significant economic and health burden on individuals and societal development.^1^ According to the International Diabetes Federation (IDF) Diabetes Atlas released in 2021, approximately 537 million people aged 20–79 years worldwide have diabetes, a figure projected to rise to 783 million by 2045.^2^ The proportion of undiagnosed diabetes cases remains alarmingly high, at around 45%.^3^ DM can be categorized into three types: gestational diabetes, type 1 diabetes (T1DM), and type 2 diabetes (T2DM). Among these, T2DM is the most prevalent, representing 90% of diabetes cases globally.^4^ With changes in lifestyle and dietary habits, the prevalence of diabetes in China has continuously increased, rising from less than 1% in 1980^5^ to 12.4% in 2018.^6^ Currently, China has the highest number of diabetes patients in the world, accounting for over a quarter of the global diabetes population. There are approximately 140 million patients aged 20–79 years old in China.^2^

T2DM is generally associated with urbanization, aging, overweight, obesity, and genetic susceptibility.^7^ Overweight and obesity are the primary risk factors.^8^, ^9^ As China's economy develops, lifestyle and dietary patterns have changed, leading to an increasing prevalence of obesity,^10^ which is expected to result in a continued rise in the diabetes burden. Additionally, there is a trend toward younger onset of diabetes, with prevalence rates of 5.0% in the 18–29‐year age group, 6.5% in the 30–39‐year age group, and 11.1% in the 40–49‐year age group.^6^ The increasing incidence among the working‐age population severely impacts societal productivity. Moreover, awareness, treatment, and control rates of diabetes in China are relatively low.^6^ The large number of individuals in the prediabetes stage indicates that prevalence could continue to rise. In 2019, China saw nearly 4 million new cases of diabetes and over 170 000 diabetes‐related deaths.^11^

Therefore, this study utilized the Global Burden of Disease (GBD) database to comprehensively analyze the incidence, mortality, and disease burden of diabetes in China from 1990 to 2021. The aim of this study was to fully understand the diabetes prevention and control situation in China and provide a basis for improving diabetes prevention and control efforts in the future.

## 2 METHODS

### 2.1 Study population and data collection

Our analysis of the GBD Study 2021 utilized repeated cross‐sectional data from the Global Health Data Exchange. This comprehensive dataset covers the global burden of 288 causes of death, 371 diseases and injuries, and 88 risk factors, including T1DM and T2DM, across 21 regions and 204 countries and territories from 1990 to 2021. The study population included individuals diagnosed with any form of diabetes, T1DM, or T2DM in China.^12^

From the GBD Study 2021, we extracted information on diabetic diseases in China, focusing on sex‐ and age‐specific incidence, prevalence, and disability‐adjusted life years (DALYs). This includes DALYs, years lived with disability (YLDs), and years of life lost (YLLs) attributable to each level 2 risk factor, with corresponding 95% uncertainty intervals (UIs). DALYs reflect the potential reduction in disease burden if population exposure to specific risk factors is altered. After adjusting for comorbidity, microsimulation was used to estimate YLDs, while YLLs were calculated by multiplying the estimated number of diabetes‐related deaths by the standard life expectancy at the age of death. The sum of YLDs and YLLs provided the DALYs. Detailed methodology for the GBD Study 2021 is available in other sources.^13^, ^14^

In the GBD Study, diabetes is defined as a doctor‐diagnosed disease identified through a diabetic registry or hospital records. To estimate the nonfatal and fatal burdens of T1DM and T2DM, along with complications, a Bayesian meta‐regression modeling tool, DisMod‐MR 2.1, was used. This tool analyzed and estimated various disease parameters, their epidemiological relationships, and geospatial relationships to produce prevalence and incidence estimates.^15^ Nonfatal and fatal outcomes in individuals with diabetes, including complications, were also estimated. The study extrapolated and modeled data based on reported cases of diabetes, juvenile onset diabetes, and insulin‐dependent diabetes, making the database applicable to most countries without age restrictions. Spatiotemporal Gaussian process regression was used to model input data in areas lacking complete datasets, providing smoothing over age, time, and location.^15^, ^16^

In this study, we collected data on T2DM and T1DM in China, encompassing 21 age groups (all ages, <5 years, 5–9 years, 10–14 years, 15–19 years, 20–24 years, 25–29 years, 30–34 years, 35–39 years, 40–44 years, 45–49 years, 50–54 years, 55–59 years, 60–64 years, 65–69 years, 70–74 years, 75–79 years, 80–84 years, 85–89 years, 90–94 years, ≥95 years) for both men and women.

### 2.2 Statistical Analysis

The age‐standardized rates (ASR) were calculated using the GBD world population age standard as a reference.^17^, ^18^ Direct standardization yields age‐adjusted rates, which are weighted averages of age‐specific rates, representing the relative age distribution. The ASR is calculated using the following equation:

$$\mathrm{ASR}=\frac{\sum_{i=1}^{A}a_{i}\omega_{i}}{\sum_{i=1}^{A}\omega_{i}}\times 100\;\;000,$$

where $a_{i}$ and $\omega_{i}$ represent age‐specific rates and the number of persons (or weight) in the same age subgroup of the chosen reference standard population (where denotes the age class), respectively. The estimated annual percentage changes (EAPC) and its 95% confidence interval (CI) are calculated using a log‐linear regression as follows: Ln (ASR) = *α* + *βx* + *ε*, where *x* is the year and *β* is the regression coefficient. EAPC was determined as 100 × [exp(*β*) − 1], and the 95% CI of EAPC were derived from the standard error produced by the log‐linear regression. The ASR was considered to have increased if the EAPC and its 95% CI were >0, decreased if they were <0, and relatively stable if the 95% CI included 0. Population‐attributable fractions (PAF) were calculated for each risk factor. Percentages and the number of DALYs are not mutually exclusive.^19^ The sum of the PAF for the risk factors may exceed 100% because many of these risk factors have effects that are partly or wholly mediated through another risk factor or factors.^14^

## 3 RESULTS

### 3.1 Overall diabetes burden

In 2021, the absolute number of new DM cases in China was estimated at 4003543.82 (95% UI: 3603717.14–4441554.24), corresponding to an ASR of 244.57 per 100 000 population (95% UI: 223.72–266.48). The estimated EAPC from 1990 to 2021 was 1.10 (95% CI: 1.02–1.19). For T1DM, the incidence was 32057.58 cases (95% UI: 26799.24–39554.53) with an ASR of 2.67 per 100 000 population (95% UI: 2.21–3.26) and an EAPC of 1.24 (95% CI: 1.14–1.34). T2DM accounted for 3971486.24 new cases (95% UI: 3572722.5–4410204.53) with an ASR of 241.9 per 100 000 population (95% UI: 221.02–263.71) and an EAPC of 1.10 (95% CI: 1.01–1.19).

The total prevalence of DM in 2021 was 117288553.93 cases (95% UI: 107649694.48–128071007.53), corresponding–an ASR of 6142.29 per 100 000 population (95% UI: 5601.11–6704.38), with an EAPC of 1.65 (95% CI: 1.55–1.74). For T1DM, the prevalence was 1442775.09 cases (95% UI: 1173170.34–1779498.9) with an ASR of 86.78 per 100 000 population (95% UI: 70.55–107.44) and an EAPC of 1.27 (95% CI: 1.18–1.38). The prevalence of T2DM was 115845778.84 cases (95% UI: 106137169.52–126653486.6) with an ASR of 6055.51 per 100 000 population (95% UI: 5510.07–6614.27) and an EAPC of 1.65 (95% CI: 1.56–1.75).

In 2021, there were 178475.73 deaths (95% UI: 147957.14–211654.89) caused by DM, with an age‐standardized mortality rate of 8.98 per 100 000 population (95% UI: 7.45–10.61). The EAPC for DM‐related deaths was −0.33 (95% CI: −0.59 to −0.08). Deaths from T1DM were estimated at 3960.35 (95% UI: 3122.49–5010.94), with an ASR of 0.23 per 100 000 population (95% UI: 0.19–0.29) and an EAPC of −3.15 (95% CI: −3.41 to −2.88). For T2DM, there were 174515.38 deaths (95% UI: 144849.6–207116.82), with an ASR of 8.74 per 100 000 population (95% UI: 7.26–10.35) and an EAPC of −0.22 (95% CI: −0.49 to 0.04).

The DALYs due to DM in 2021 were 11713613.86 (95% UI: 9046221.56–15013009.95), with an ASR of 585.43 per 100 000 population (95% UI: 448.94–754.32) and an EAPC of 0.57 (95% CI: 0.42–0.72). For T1DM, the DALYs were 248595.99 (95% UI: 201970.05–302828.08), with an ASR of 15.59 per 100 000 population (95% UI: 12.75–18.82) and an EAPC of −2.29 (95% CI: −2.47 to −2.1). T2DM accounted for 11465017.87 DALYs (95% UI: 8834150.96–14700712.03), with an ASR of 569.84 per 100 000 population (95% UI: 435.43–734.18) and an EAPC of 0.69 (95% CI: 0.54–0.84, Table 1).

**TABLE 1:** Absolute number and age‐standardized rates per year of incident and prevalent diabetes, deaths from diabetes and DALYs due to diabetes in 2021, and estimated annual percentage changes (EAPC) in China for 1990–2021, by types of diabetes.

Types	Absolute number in 2021	Age‐standardized rates in 2021	EAPC (95% CI)
Incidence (95% UI)			
Diabetes mellitus	4003543.82 (3603717.14 to 4441554.24)	244.57 (223.72 to 266.48)	1.10 (1.02 to 1.19)
Diabetes mellitus type I	32057.58 (26799.24 to 39554.53)	2.67 (2.21 to 3.26)	1.24 (1.14 to 1.34)
Diabetes mellitus type II	3971486.24 (3572722.5 to 4410204.53)	241.90 (221.02 to 263.71)	1.10 (1.01 to 1.19)
Prevalence (95% UI)			
Diabetes mellitus	117288553.93 (107649694.48 to 128071007.53)	6142.29 (5601.11 to 6704.38)	1.65 (1.55 to 1.74)
Diabetes mellitus type I	1442775.09 (1173170.34 to 1779498.9)	86.78 (70.55 to 107.44)	1.27 (1.18 to 1.38)
Diabetes mellitus type II	115845778.84 (106137169.52 to 126653486.6)	6055.51 (5510.07 to 6614.27)	1.65 (1.56 to 1.75)
Deaths (95% UI)			
Diabetes mellitus	178475.73 (147957.14 to 211654.89)	8.98 (7.45 to 10.61)	−0.33 (−0.59 to −0.08)
Diabetes mellitus type I	3960.35 (3122.49 to 5010.94)	0.23 (0.19 to 0.29)	−3.15 (−3.41 to −2.88)
Diabetes mellitus type II	174515.38 (144849.6 to 207116.82)	8.74 (7.26 to 10.35)	−0.22 (−0.49 to 0.04)
DALYs (95% UI)			
Diabetes mellitus	11713613.86 (9046221.56 to 15013009.95)	585.43 (448.94 to 754.32)	0.57 (0.42 to 0.72)
Diabetes mellitus type I	248595.99 (201970.05 to 302828.08)	15.59 (12.75 to 18.82)	−2.29 (−2.47 to −2.1)
Diabetes mellitus type II	11465017.87 (8834150.96 to 14700712.03)	569.84 (435.43 to 734.18)	0.69 (0.54 to 0.84)

Abbreviations: CI, confidence interval; DALYs, disability‐adjusted life years; EAPC, estimated annual percentage changes; UI, uncertainty intervals.

### 3.2 The burden of diabetes mellitus by age and sex

In 2021, the DALYs due to DM in China exhibited notable trends across different age groups and sexes. For males, both the number and rate of DALYs increased consistently with age, peaking in the 65–69‐year age group. The DALYs continued to rise through early adulthood and middle age, showing the highest values in late adulthood before slightly declining in the oldest age groups. Specifically, DALYs numbers rose from 1316.32 (95% UI: 930.62–1798.82) in the <5 years group to 803249.83 (95% UI: 628219.14–1021944.41) in the 65–69 years group. DALYs rates increased from 3.16 per 100 000 (95% UI: 2.24–4.32) in the <5 years group to 4496.14 per 100 000 (95% UI: 3719.79–5307.87) in the 90–94 years group. For females, the trend was somewhat similar, with DALYs numbers and rates increasing with age, but with some fluctuations in older age groups. The DALYs peaked in the 65–69‐year age group and showed a slight decrease afterward. DALYs numbers increased from 1870.69 (95% UI: 1150.08–3007.35) in the <5 years group to 839864.06 (95% UI: 651929.79–1049262.39) in the 65–69 years group.

**FIGURE 1:**
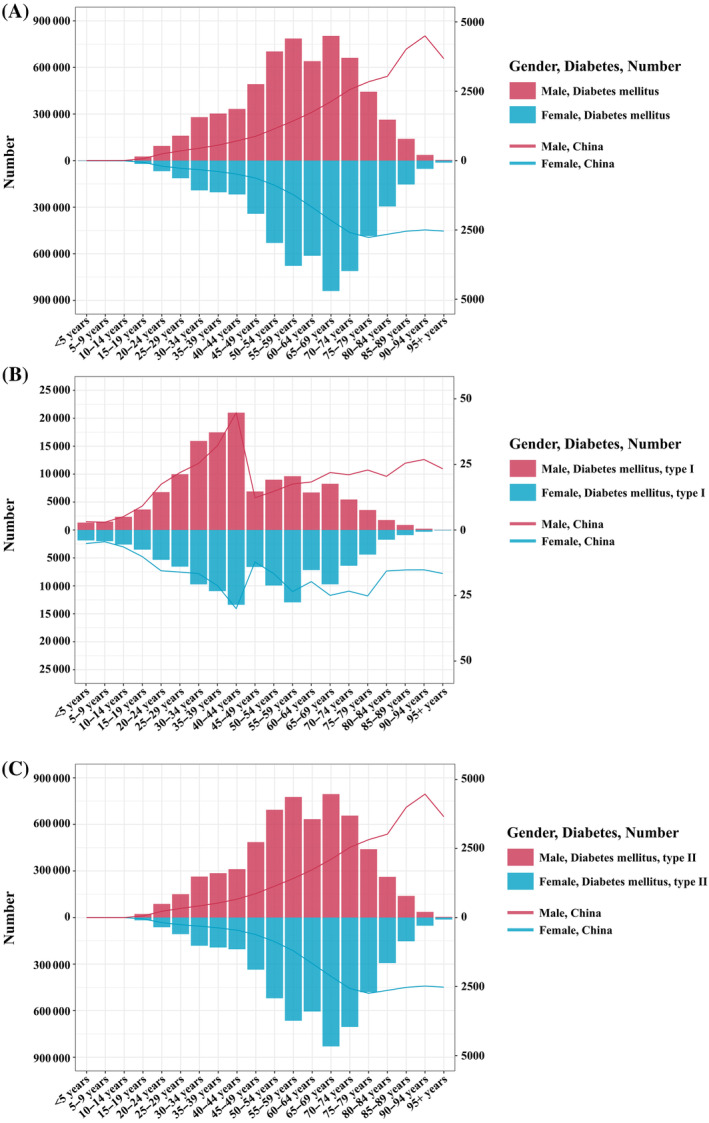
The absolute number and age‐standardized disability‐adjusted life years (DALYs) (per 100 000 people per year) in China in 2021 for both sexes and all ages. (A) Diabetes mellitus; (B) diabetes mellitus I; (C) diabetes mellitus II.

DALYs rates increased from 5.19 per 100 000 (95% UI: 3.19–8.35) in the <5 years group to 2770.69 per 100 000 (95% UI: 2216.15–3297.52) in the 75–79 years group. Overall, males consistently exhibited higher DALYs compared with females across all age groups. Both sexes showed an increase in DALYs with age, with the highest burden observed in late adulthood, indicating a greater impact of DM on older adults (Figure 1A).

In 2021, the DALYs due to T1DM in China exhibited notable trends across different age groups and sexes. For males, both the number and rate of DALYs increased consistently with age, peaking in the 40–44‐year age group. The DALYs continued to rise through early adulthood and middle age, showing the highest values in middle adulthood before slightly declining in the oldest age groups. Specifically, DALYs numbers rose from 1316.32 (95% UI: 930.62–1798.82) in the <5 years group to 20981.91 (95% UI: 15356.40–27591.29) in the 40–44 years group. DALYs rates increased from 3.16 per 100 000 (95% UI: 2.24–4.32) in the <5 years group to 44.71 per 100 000 (95% UI: 32.73–58.80) in the 40–44 years group. For females, the trend was somewhat similar, with DALYs numbers and rates increasing with age, but with some fluctuations in older age groups. The DALYs peaked in the 40–44‐year age group and showed a slight decrease afterward. DALYs numbers increased from 1870.69 (95% UI: 1150.08 to 3007.35) in the <5 years group to 13388.68 (95% UI: 10034.58–18006.38) in the 40–44 years group. DALYs rates increased from 5.19 per 100 000 (95% UI: 3.19–8.35) in the <5 years group to 30.01 per 100 000 (95% UI: 22.49–40.37) in the 40–44 years group. Overall, males consistently exhibited higher DALYs compared with females across all age groups. Both sexes showed an increase in DALYs with age, with the highest burden observed in middle adulthood, indicating a greater impact of T1DM on adults aged 40–44 years (Figure 1B).

In 2021, the DALYs due to T2DM in China exhibited notable trends across different age groups and sexes. For males, both the number and rate of DALYs increased consistently with age, peaking in the 55–59‐year age group before slightly declining in the oldest age groups. Specifically, DALYs numbers rose from 22685.32 (95% UI: 13748.73–35003.63) in the 15–19 years group to 776480.57 (95% UI: 587814.81–1000140.11) in the 55–59 years group, peaking at 794987.87 (95% UI: 621918.77–1011592.75) in the 65–69 years group. DALYs rates increased from 56.60 per 100 000 (95% UI: 34.30–87.33) in the 15–19 years group to 1414.78 per 100 000 (95% UI: 1071.02–1822.29) in the 55–59 years group, peaking at 2106.48 per 100 000 (95% UI: 1647.90–2680.42) in the 65–69 years group. For females, the trend was somewhat similar, with DALYs numbers and rates increasing with age, peaking in the 65–69‐year age group and showing a slight decrease afterward. In detail, DALYs numbers increased from 17779.52 (95% UI: 10979.60–27650.22) in the 15–19 years group to 664855.84 (95% UI: 501187.17–870530.60) in the 55–59 years group, peaking at 830141.22 (95% UI: 643875.53–1037528.99) in the 65–69 years group. DALYs rates increased from 51.40 per 100 000 (95% UI: 31.74–79.94) in the 15–19 years group to 1207.54 per 100 000 (95% UI: 910.28–1581.09) in the 55–59 years group, peaking at 2130.57 per 100 000 (95% UI: 1652.51–2662.83) in the 65–69 years group. Overall, males exhibited higher DALYs compared with females across most age groups. Both sexes showed an increase in DALYs with age, with the highest burden observed in middle and late adulthood (Figure 1C).

### 3.3 DM‐related DALYs attributable to risk factors

In 2021, various risk factors contributed significantly to the DALYs due to DM in China, affecting all ages and both sexes. The data for each risk factor includes the number of DALYs, YLDs, and YLLs, along with their respective percentages and 95% UIs.

The total number of DALYs attributed to air pollution accounted for 2274206.91 (95% UI: 1317236.09–3507096.01), with the PAF of 19.4% (95% UI: 12.1%–27%). The YLDs were 1553189.22 (95% UI: 843118.25–2518024.45), contributing 19.5% (95% UI: 12.2%–27.1%), and the YLLs were 721017.69 (95% UI: 444828.2–1050911.33), representing 19.3% (95% UI: 12%–26.7%).

The total number of DALYs attributed to nonoptimal temperature was 279084.97 (95% UI: 188877.92–406507.92), contributing the PAF of 2.4% (95% UI: 1.6%–3.6%). The YLLs attributed to this factor were 279084.97 (95% UI: 188877.92–406507.92), representing 7.5% (95% UI: 5.3%–10.4%). Data for YLDs was not available for this risk factor.

The total number of DALYs attributed to tobacco use was responsible for 2063008.65 (95% UI: 1272700.51–3020164.02), representing the PAF of 17.6% (95% UI: 11.8%–23%). The YLDs were 1440366.38 (95% UI: 846182.21–2234204.97), contributing 18.1% (95% UI: 12.1%–23.6%), and the YLLs were 622642.27 (95% UI: 389132.76–875554.96), representing 16.6% (95% UI: 10.9%–21.8%).

The total number of DALYs attributed to alcohol use accounted for 207800.22 (95% UI: 57830.31–431011.35), contributing the PAF of 1.8% (95% UI: 0.5%–3.5%). The YLDs were 139937.96 (95% UI: 30209.07–320528.87), representing 1.7% (95% UI: 0.4%–3.7%), and the YLLs were 67862.25 (95% UI: 24679.95–131693.81), contributing 1.8% (95% UI: 0.7%–3.5%).

The total number of DALYs attributed to dietary risks were 2702413.45 (95% UI: 337623.07–4927021.16), representing the PAF of 23% (95% UI: 2.9%–39.5%). The YLDs were 1899437.32 (95% UI: 243824.33–3606789.43), contributing 23.8% (95% UI: 3%–40.6%), and the YLLs were 802976.13 (95% UI: 92123.02–1444695.88), representing 21.4% (95% UI: 2.6%–37.2%).

The total number of DALYs attributed to low physical activity contributed to 757445.48 (95% UI: 327374.89–1219478.84), representing the PAF of 6.5% (95% UI: 2.6%–10.1%). The YLDs were 459189.62 (95% UI: 183697.54–773799.43), contributing 5.8% (95% UI: 2.3%–9%), and the YLLs were 298255.86 (95% UI: 128264.79–491864.56), representing 8% (95% UI: 3.4%–12.4%).

The total number of DALYs attributed to high body mass index (BMI), the leading risk factor, was 5898042.78 (95% UI: 2766366.44–8874173.58), representing the PAF of 50.3% (95% UI: 23.6%–70.7%). The YLDs were 4218154.6 (95% UI: 1970468.23–6496843.55), contributing 52.9% (95% UI: 25.5%–73.3%), and the YLLs were 1679888.18 (95% UI: 705017.06–2561659.56), representing 44.8% (95% UI: 19.4%–65.6%).

High fasting plasma glucose was responsible for the highest burden, with 11712640.26 (95% UI: 9049773.42–15010569.42), representing 100% (95% UI: 100%–100%). The YLDs were 7971666.36 (95% UI: 5408050.24–11189804.17), contributing 100% (95% UI: 100%–100%), and the YLLs were 3740973.89 (95% UI: 3086971.26–4464988.66), representing 100% (95% UI: 100%–100%, Table 2).

**TABLE 2:** Diabetes mellitus‐related DALYs (number and percentages) associated with risk factors and their clusters in 2021, for all ages and both sexes.

Risk factor	DALYs		YLDs		YLLs	
	Number	Percentages	Number	Percentages	Number	Percentages
Air pollution	2274206.91 (1317236.09–3507096.01)	19.4 (12.1–27)	1553189.22 (843118.25–2518024.45)	19.5 (12.2–27.1)	721017.69 (444828.2–1050911.33)	19.3 (12.0–26.7)
Nonoptimal temperature	279084.97 (188877.92–406507.92)	2.4 (1.6–3.6)	NA	NA	279084.97 (188877.92–406507.92)	7.5 (5.3–10.4)
Tobacco	2063008.65 (1272700.51–3020164.02)	17.6 (11.8–23)	1440366.38 (846182.21–2234204.97)	18.1 (12.1–23.6)	622642.27 (389132.76–875554.96)	16.6 (10.9–21.8)
Alcohol use	207800.22 (57830.31–431011.35)	1.8 (0.5–3.5)	139937.96 (30209.07–320528.87)	1.7 (0.4–3.7)	67862.25 (24679.95–131693.81)	1.8 (0.7–3.5)
Dietary risks	2702413.45 (337623.07–4927021.16)	23 (2.9–39.5)	1899437.32 (243824.33–3606789.43)	23.8 (3–40.6)	802976.13 (92123.02–1444695.88)	21.4 (2.6–37.2)
Low physical activity	757445.48 (327374.89–1219478.84)	6.5 (2.6–10.1)	459189.62 (183697.54–773799.43)	5.8 (2. 3,9)	298255.86 (128264.79–491864.56)	8.0 (3.4–12.4)
High body mass index	5898042.78 (2766366.44–8874173.58)	50.3 (23.6–70.7)	4218154.6 (1970468.23–6496843.55)	52.9 (25.5–73.3)	1679888.18 (705017.06–2561659.56)	44.8 (19.4–65.6)
High fasting plasma glucose	11712640.26 (9049773.42–15010569.42)	100 (100–100)	7971666.36 (5408050.24–11189804.17)	100 (100–100)	3740973.89 (3086971.26–4464988.66)	100 (100–100)

Abbreviations: DALYs, disability‐adjusted life years; NA, not available; YLDs, years lived with disability; YLLs, years of life lost.

### 3.4 The trends of age‐standardized PAF across the different DM types

**FIGURE 2:**
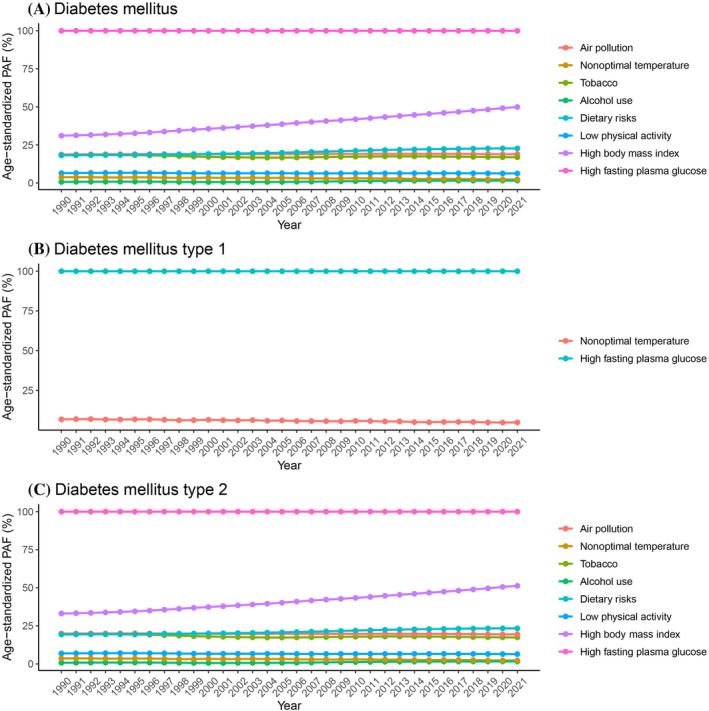
Age‐standardized population‐attributable fractions (PAF) of disability‐adjusted life years (DALYs) of risk factors in China during 1990 and 2021. (A) Diabetes mellitus; (B) diabetes mellitus I; (C) diabetes mellitus II.

In China, the DALYs attributable to diabetes caused by high fasting plasma glucose remained consistently high from 1990 to 2021, with the percentage holding steady at 99.99%. This indicated that high fasting plasma glucose has been a major contributing factor to the diabetes burden throughout this period. Air pollution was another significant factor. The percentage of diabetes burden due to air pollution decreased from 18.71% in 1990 to 18.92% in 2021. Although there have been fluctuations, the overall trend showed a slight decrease, suggesting improvements in air quality over the years. Tobacco use was a notable risk factor for diabetes. The data showed a decline in the diabetes burden due to tobacco use, from 18.24% in 1990 to 16.92% in 2021. This decline can be attributed to better public health policies and increased antismoking campaigns. The DALYs attributable to diabetes caused by dietary risks increased from 18.18% in 1990 to 22.69% in 2021. This upward trend reflected the growing impact of dietary habits and nutritional intake on the diabetes burden. Low physical activity was a significant risk factor. The percentage of diabetes burden due to low physical activity changed slightly, from 6.46% in 1990 to 6.26% in 2021, indicating a consistent impact of physical inactivity on the diabetes burden. The impact of alcohol use on the diabetes burden was relatively small but noteworthy. The percentage increased from 0.69% in 1990 to 1.58% in 2021, suggesting a rising influence of alcohol consumption on the diabetes burden. High BMI is a major risk factor for diabetes. The data showed a significant increase in the percentage of diabetes burden due to high BMI, from 31.08% in 1990 to 49.90% in 2021, reflecting the escalating obesity problem in China. The impact of nonoptimal temperature on the diabetes burden was relatively minor but still relevant. The percentage decreased from 3.78% in 1990 to 2.33% in 2021, possibly due to climate change and improved environmental control measures (Figure 2A).

The burden of T1DM due to high fasting plasma glucose remained stable from 1990 to 2021, with percentages ranging from 99.96% to 99.98%. Additionally, the impact of nonoptimal temperature on T1DM decreased from 6.77% in 1990 to 4.85% in 2021 (Figure 2B).

The burden of T2DM is influenced by various risk factors, including tobacco use, air pollution, dietary risks, low physical activity, nonoptimal temperature, and high BMI. Notably, the percentage of T2DM burden due to high BMI increased significantly from 33.09% in 1990 to 51.28% in 2021, highlighting the critical role of obesity in the prevalence of T2DM (Figure 2C).

### 3.5 The ranking levels of risk factors across age groups

**FIGURE 3:**
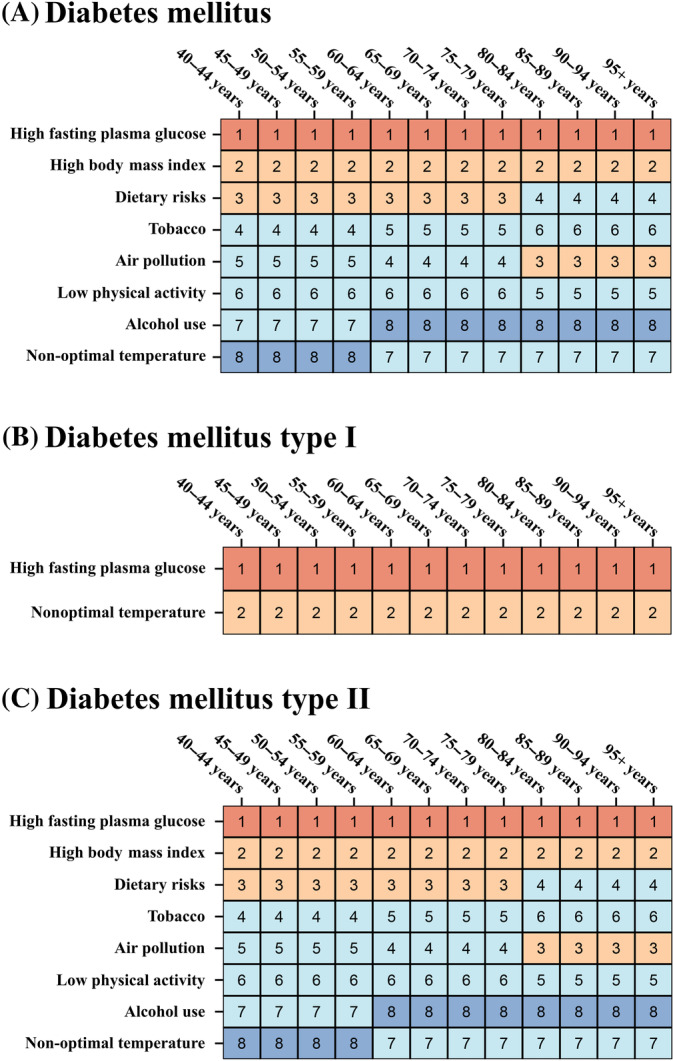
The ranking level of diabetes mellitus‐related disability‐adjusted life years (DALYs) attributable to risk factors by specific age group, for both sexes, 2021, China. (A) diabetes mellitus; (B) diabetes mellitus I; (C) diabetes mellitus II. Numbers show the ranking level (1 = highest, 15 = lowest) by the number of DALYs attributable to the corresponding risk factors. Red shows 1st ranking; light brown, second and third ranking; very light yellow, 4–7 ranking; very light blue, 8–13 ranking; and dark blue, 14–15 ranking.

For diabetes, from ages 40–95, the top two risk factors for DALYs numbers are high fasting plasma glucose and high BMI. From ages 40 to 79, the third‐ranked risk factor is Dietary Risks, whereas after age 79, the third‐ranked risk factor is air pollution (Figure 3A). For T1DM, the highest risk factor remains high fasting plasma glucose, followed by nonoptimal temperature (Figure 3B). The ranking levels of risk factors for DALYs number attributed to T2DM is similar to that of overall DM (Figure 3C).

### 3.6 The comparison of DALYs by age and sex

In China, the number of DALYs of DM attributed to risk factors was various based on specific age and sex. For both males and females aged 40–44, high fasting plasma glucose accounts for approximately 550 385 DALYs number in males and 299 972 DALYs number in females, making it the leading risk factor. High BMI contributed to around 330 341 in males and 195 010 in females. Tobacco use ranked as a significant risk factor for males (116 664) but was less impactful for females (47 736).

Among individuals aged 45–49 years, high fasting plasma glucose remained the dominant risk factor, accounting for 834 780 DALYs number in males and 463 486 DALYs number in females. High BMI was the second highest, with 513 393 DALYs number in males and 309 894 DALYs number in females. Dietary risks contributed notably as the third risk factor, with 219 192 DALYs number in males and 172 848 in females.

In the age group 50–54 years, high fasting plasma glucose significantly impacted males (1 233 090 DALYs number) and females (697 882 DALYs number). High BMI also remained critical, contributing 732 959 DALYs number in males and 468 962 DALYs number in females. Tobacco use and dietary risks were prominent, with tobacco causing 263 864 DALYs number in males and 124 568 DALYs number in females, and dietary risks contributing 308 018 DALYs number in males and 246 848 DALYs number in females.

For ages 55–59, high fasting plasma glucose impacted 1 463 777 DALYs number in males and 885 436 DALYs number in females. High BMI remained substantial, with 817 454 DALYs number in males and 582 796 DALYs number in females. The third‐ranked dietary risks accounted for 351 632 DALYs number in males and 307 315 DALYs number in females.

At ages 60–64, high fasting plasma glucose was responsible for 1 252 962 DALYs number in males and 776 624 DALYs number in females. High BMI contributed to 665 808 DALYs number in males and 507 388 in females. Air pollution became more significant, accounting for 250 039 DALYs number in males and 186 696 DALYs number in females.

For those aged 65–69, high fasting plasma glucose remained dominant, with 1 643 004 DALYs number in males and 1 049 584 DALYs number in females. High BMI and air pollution followed, with high BMI contributing 800 569 DALYs number in males and 668 697 DALYs number in females, and air pollution accounting for 328 227 DALYs number in males and 253 958 DALYs number in females.

In the 70–74‐year age group, high fasting plasma glucose impacted 1 373 114 DALYs number in males and 866 558 DALYs number in females. High BMI contributed to 592 378 DALYs number in males and 521 279 DALYs number in females, with air pollution following closely behind, accounting for 274 972 in males and 211 454 in females.

Among individuals aged 75–79, high fasting plasma glucose remained significant with 928 612 DALYs number in males and 578 942 DALYs number in females. High BMI and air pollution also impacted, contributing 342 820 in males and 318 341 in females for high BMI, and 185 928 in males and 142 987 in females for air pollution.

For those aged 80–84, high fasting plasma glucose was responsible for 558 675 DALYs number in males and 355 463 DALYs number in females. High BMI and air pollution followed, contributing 163 295 in males and 164 512 in females, and 112 017 in males and 87 972 in females for air pollution.

In the 85–89‐year age group, high fasting plasma glucose remained significant, with 293 699 DALYs number in males and 184 492 DALYs number in females. High BMI and air pollution continued to be significant, contributing 86 428 in males and 85 666 in females, and 58 869 in males and 45 797 in females for air pollution.

**FIGURE 4:**
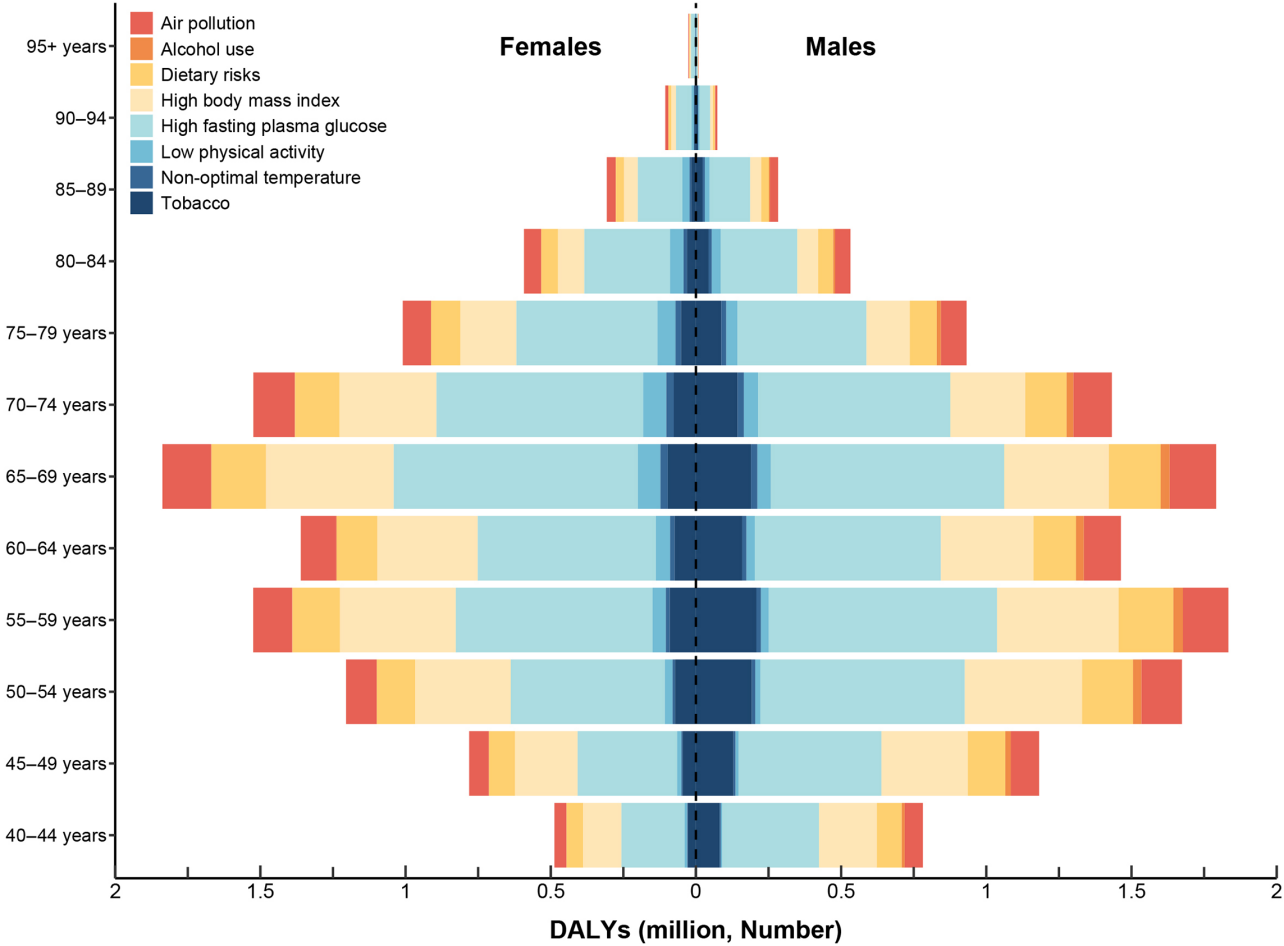
The number of diabetes mellitus‐related disability‐adjusted life years (DALYs) attributable to risk factors by specific age group and sex, 2021, China.

For those aged 90–94, high fasting plasma glucose remained the leading risk factor with 89 394 DALYs number in males and 63 172 DALYs number in females. High BMI and air pollution followed, contributing 26 550 in males and 29 763 in females, and 17 905 in males and 15 886 in females for air pollution. In the oldest age group of 95+, high fasting plasma glucose still led, accounting for 17 604 in males and 15 928 in females. High BMI and air pollution also remained significant, contributing 5303 in males and 7541 in females, and 3518 in males and 3926 in females for air pollution **(**Figure 4).

## 4 DISCUSSION

Our analysis of data from the most recent GBD study revealed that in 2021, the incidence, prevalence, deaths, and DALYs attributable to DM in China were 4003543.82, 117288553.93, 178475.73, and 11713613.86, respectively. Corresponding ASR were 244.57, 6142.29, 8.98, and 585.43 per 100 000 population, respectively. The EAPC results indicated that from 1990 to 2021, the age‐standardized incidence rate, prevalence rate, and DALYs rate for DM, T1DM, and T2DM showed an overall increasing trend over the past three decades. This aligns with previous research findings^20^, ^21^ and can be attributed to factors such as improved economic and social development, extended survival periods for chronic diseases like hypertension and hyperlipidemia, and unhealthy lifestyles, including smoking, alcohol consumption, and prolonged sedentary behavior.^22^, ^23^, ^24^ Additionally, the aging population in China is a significant driver of the increased incidence and prevalence rates of DM.^25^ However, the age‐standardized mortality rate for DM has shown a declining trend, suggesting a reduced risk of diabetes‐related mortality in China. This may be linked to improved access to healthcare services and advancements in medical care in recent years.^26^ Diabetes is characterized by high prevalence and disability rates, leading to severe complications affecting the heart, blood vessels, eyes, kidneys, and nerves.^27^ The treatment costs for patients with complications are significantly higher than for those without.^28^ Despite this, studies have shown that the awareness rate of diabetes in China is only 36.5%, the treatment rate is 32.2%, and the control rate among those treated is 49.2%.^29^ Therefore, it is crucial to enhance public awareness of self‐management of blood glucose levels, emphasize early diagnosis of diabetes, improve the quality of community diabetes management, reduce the incidence of diabetes complications, and alleviate the disability burden caused by diabetes.

Regarding the types of diabetes, T2DM constitutes the major burden of diabetes in China.^30^ T2DM primarily occurs in adulthood, with obesity being a significant risk factor.^31^ Studies have shown that regular moderate‐intensity exercise can effectively prevent the onset of T2DM.^32^ Furthermore, engaging in physical activity can influence blood glucose levels in the body and reduce the risk of developing diabetes.^33^

From a gender perspective, between 1990 and 2021, Chinese males exhibited higher DALYs than females across most age groups. This disparity may be attributed to the higher exposure levels to risk factors such as obesity, smoking, excessive alcohol consumption, and sedentary behavior among males.^22^, ^34^, ^35^ In terms of age distribution, the high‐incidence period for DALYs due to DM and T2DM in China in 2021 was between the ages of 50 and 74. The number and rate of DALYs increased continuously with age, peaking in the 65–69‐year age group, indicating a greater impact of DM and T2DM on older adults. This finding is consistent with the results of most previous studies.^36^, ^37^ For T1DM, the highest DALYs burden occurred in middle age, particularly affecting adults aged 40–44 years. Our results suggest that health monitoring should be intensified for middle‐aged and older populations, with a particular focus on promoting health awareness among middle‐aged and older males, and encouraging regular blood glucose checks in this demographic.

An analysis of DM risk factors revealed that the DALYs due to DM in China were significantly impacted by various risk factors. The leading risk factors included high fasting plasma glucose, high BMI, dietary risks, air pollution, tobacco, low physical activity, nonoptimal temperature, and alcohol consumption. Among these, DM‐related DALYs due to high fasting plasma glucose consistently remained at a high level of 99.99%. The DALYs associated with dietary risks, alcohol consumption, and high BMI showed an increasing trend, whereas those related to air pollution, tobacco, low physical activity, and nonoptimal temperature were on the decline.

Overweight and obesity, defined as high BMI, are major risk factors for T2DM, aligning with the findings of many other studies.^38^, ^39^ This is attributed to the significantly higher relative risk (RR) of T2DM in individuals who are overweight or obese compared to those with a normal BMI.^40^ Research indicates that adipocytes can be considered a type of endocrine cell, and adipose tissue functions as an endocrine organ. Adipocytes secrete various adipokines and cytokines, which can increase diabetes risk through multiple pathways, such as enhancing insulin resistance.^41^ Therefore, controlling body weight is crucial for curbing the prevalence of T2DM. Among males, T2DM cases attributable to high BMI peaked in the 55–59‐year age group, while in females, the peak occurred in the 65–69‐year age group, highlighting the importance of preventing overweight and obesity in middle‐aged and older populations. Moreover, numerous prospective studies have confirmed that individual dietary factors play a significant role in preventing T2DM.^42^, ^43^ Consuming more whole grains and leafy green vegetables, and less refined grains, processed meats, red meats, and sugar‐sweetened beverages can reduce the risk of diabetes onset.^36^, ^44^ From 1990 to 2021, the burden of T2DM attributed to dietary factors among Chinese residents remained stable at around 23%. Recently, dietary factors have been shown to be the second most significant contributor to T2DM burden after overweight or obesity, and they may also indirectly increase T2DM risk by contributing to overweight or obesity.^45^ Additionally, our study showed that the proportion of T2DM cases attributed to low physical activity remains relatively stable across all age groups for both males and females, indicating that engaging in physical exercise at any age can effectively prevent T2DM. Numerous studies have demonstrated that regular physical activity improves insulin sensitivity, glycemic control, and metabolic status in both diabetic and nondiabetic individuals.^46^, ^47^ The burden of T2DM caused by smoking is predominantly borne by males. Smoking leads to insulin resistance or compensatory insulin secretion deficiency through various mechanisms, and nicotine in cigarettes may exert a direct toxic effect on cellular function.^48^, ^49^ Therefore, reducing the smoking population has significant potential to decrease the incidence of T2DM. Furthermore, industrialization in developing stages has exacerbated environmental pollution, playing a “catalyst” role in the diabetes incidence process. Meroni et al.^50^ found that for every unit increase in PM_10_ or NO_2_ concentration, the diabetes prevalence increased by 0.81% and 0.41%, respectively. Air pollutants such as PM_2.5_, PM_2.5–10_, NO_2_, and NO synergistically contribute to diabetes in obese individuals.^51^ These pollutants can increase insulin resistance and T2DM incidence.^52^, ^53^ The aforementioned studies indicate that prolonged exposure to atmospheric particulate matter and air pollutants will lead to an increased incidence of T2DM.

This study has several limitations. First, the GBD 2021 dataset provides information at the national level only, lacking detailed data at the provincial and municipal levels. Second, China's social structure is distinctly urban–rural, but due to data limitations, we could not analyze the burden of DM separately for urban and rural areas. Third, the GBD 2021 does not capture the severity of DM, its economic burden, or the progression of its comorbidities. Fourth, the projections are based on simulations under specific conditions, while the occurrence of diseases is influenced by many uncontrollable factors, including demographics, environment, healthcare, and economic aspects. Consequently, the predicted results may deviate from actual outcomes, and the accuracy of the predictions will decrease over time. Therefore, it is necessary to further refine the EAPC model to improve the accuracy of the forecasts.

## 5 CONCLUSIONS

In summary, the incidence, prevalence, and DALYs rate of diabetes in China are at relatively high levels, with a severe and rapidly growing burden of disability, particularly prominent among males. Interventions should be strengthened for major factors contributing to the diabetes burden, including high fasting plasma glucose, high BMI, air pollution, and dietary risks. Effective treatment and management of diabetes patients are essential to reduce diabetes‐related mortality and improve patients' quality of life. It is crucial to prepare for the long‐term management of the diabetes burden.

## AUTHOR CONTRIBUTIONS

*Conceptualization*: W.D and D.L; *Data curation*: W.D, L.Z., C.C., Z.R. and D.L.; *Formal analysis*: W.D., Y.J., and J.Q.; *Writing‐original draft*: W.D; *Writing‐review & editing*: W.D and D.L.

## FUNDING INFORMATION

This work was supported by Natural Science Foundation of Chongqing (Grant No. CSTB2022NSCQ‐MSX1573), Scientific and Technological Research Program of Chongqing Municipal Education Commission (Grant No. KJQN202300130).

## CONFLICT OF INTEREST STATEMENT

The authors declare no conflicts of interest.

## Data Availability

The datasets used and/or analyzed during the current study are available from the corresponding author on reasonable request.
